# Systematically testing the effects of promotion techniques on children’s fruit and vegetables intake on the long term: a protocol study of a multicenter randomized controlled trial

**DOI:** 10.1186/s12889-019-7952-1

**Published:** 2019-11-27

**Authors:** Frans Folkvord

**Affiliations:** 1 Tilburg School of Humanities and Digital Sciences, Department Communication and Cognition, Warrandelaan 2, 5037 AB Tilburg, the Netherlands; 2 From Open Evidence Research, Barcelona, Spain

**Keywords:** Fruit and vegetables, Eating behavior, Children, Food marketing, Primary schools

## Abstract

**Background:**

Eating a diet rich in fruit and vegetables is essential for healthy development, protects against chronicle diseases, and increases mental well-being. Numerous studies have consistently shown that children do not consume enough fruit and vegetables, especially among children from low socioeconomic status, while foods high in fat, sugar and salt are over-consumed. In order to improve children’s eating behavior, there is an urgent need to systematically test novel and effective methods to make fruit and vegetables more appealing and increase the intake among children. Therefore, the main aim of the proposed project is to test if food promotion techniques increase children’s fruit and vegetable intake, both on the short- and long-term.

**Methods:**

Three studies will be conducted. First, to develop the vlogs in co-creation, multiple focus groups will be held with (1) children (*N* = 25, between 8 and 13 years), (2) parents (*N* = 10), (3) vloggers (*N* = 5), and (4) fruit and vegetable producers and marketers (N = 5). Second, a multicenter randomized clinical trial will be conducted among 10 primary schools. A mixed repeated measure design with three different conditions will be used: (1) control, (2) a vlog unboxing fruit and vegetables (preparing and tasting), and (3) a vlog doing a challenge with the fruit and vegetables (e.g., contests, tricks, games). Children between 7 and 13 will participate in the experiments (*N* = 350). Third, after 6 and 12 months follow-up measurements will take place.

**Discussion:**

HFSS foods have higher intrinsically rewarding properties that make them more “wanted” and “liked” than fruit and vegetables, thereby inducing unhealthy eating behavior among children. Additionally, promotion for HFSS foods is omnipresent and increases the rewarding value of these foods. Moreover, some studies showed that the promotion of fruit and vegetables affects the intake, although a recent systematic review shows that evidence is inconclusive and a theoretical understanding for the underlying mechanism is missing. The current study aims to improve the existing knowledge by experimentally testing a newly developed theoretical model.

**Trial registration:**

Netherlands Trial registration**:** NL8077, received on 12 October 2019.

## Background

Eating a diet rich in fruit and vegetables is essential for growth and development [1], protects against many chronic diseases [2] and increases mental well-being [3–9]. Numerous studies have consistently shown that dietary intake patterns of children do not meet (inter) national dietary standards (e.g., insufficient fruit and vegetable intake, overconsumption of foods high in fat, sugar and salt [HFSS]), especially children from low socioeconomic statuses [10–13]. For example, a recent report [13] has shown that less than 25% of Dutch children between 8 and 12 years eats the recommended amount of fruit, and only 1–8% the recommended amount of vegetables.

An effective mechanism to increase consumption of food is through food promotion (the communication to the consumer through a range of marketing techniques in order to add value to a food product and persuade to consume) [14–17]. Food companies frame their messages to prime children to focus on the hedonic aspects of HFSS foods [17–23]. Considering the effectiveness and success of food promotions of HFSS foods [19, 20], it is highly relevant to examine the effectiveness of food promotion of healthier foods. Until now, studies have shown that it is quite challenging to improve the intake of fruit and vegetables among children [24]. A systematic review [24] showed that the promotion of healthy foods is highly promising and there is mounting evidence for its effectiveness, although highly depending on moderating factors [20, 25]. Meanwhile, a theoretical and systematically investigation is missing, which is necessary to improve scientifically understanding regarding the effectiveness of promoting healthier foods.

Fruits and vegetables are less intrinsically rewarding than HFSS foods, and miss therefore specific motivational capacities and incentive qualities to automatically increase the willingness to obtain these foods [26–29]. Additionally, the food industry spends incessant, sophisticated and personalized advertising to effectively increase the hedonic and rewarding value of HFSS foods, by modifying attitudes, emotions, intentions, and ultimately consumption behavior [16–20, 30–33]. Until now, most studies focused on decreasing the reinforcing value of HFSS foods, while the current study will investigate the potential of reinforcing values of healthier foods. As children age, they increasingly make their own food choices [34–38] and develop eating habits that will continue over the years and contribute to long-term health and chronic disease risks [34–38], making it urgently to improve dietary intake among children.

A healthy food promotion intervention will be developed with vlogging as the main promotional method, and actual fruit and vegetable intake among children will be measured repeatedly. Vlogs (video-blogs) have been described as short user-generated videos, distributed online where others may view, subscribe, or comment on them [39]. Vloggers increasingly promote HFSS foods by unboxing (opening and trying the promoted foods) or by a contest (e.g., tricks, games), while the effect on consumption hardly has been investigated. Vlogging is the most recent and highly popular food promotion technique targeting children [23]. Moreover, the content of vlogs can easily be modified so different forms of content among different groups of children can be tested [25].

The current study is highly original and innovative as it is the first that systematically examines the influence of food promotion on actual intake of fruit and vegetables [24]. In addition, it assesses repeatedly intake both on the short- and long-term, validates a newly developed theoretical framework to establish the underlying mechanism, focuses on vlogs as a persuasive instrument, and will take into account intensively on individual susceptibility factors.

### Theoretical framework

Recently an overarching theoretical model has been developed through an eclectic synthesis of existing theoretical models from different disciplines and recent empirical evidence has been conducted that explains and predicts whether, how, when, and for whom food promotion techniques increase children’s fruit and vegetables intake, both on the short- and long-term [40]. The four foundational assumptions of the theoretical model, *The Promotion of Fruit and Vegetables Model* are that (1) by increasing the reinforcing value of fruit and vegetables (e.g., liking and wanting) through effective food promotion techniques, (2) a reciprocal relation with eating behavior occurs, that in time (3) leads to a normalization of intake of fruit and vegetables (habitual formation). Furthermore, (4) individual and contextual factors (e.g., BMI [41, 42], SES [12, 13], Food Fussiness [43, 44], Parental Feeding Style [45, 46]) determine individual susceptibility to food promotion. In the next section, I will explain the model and associated hypotheses.

### Vlogs to reinforce the value and increase intake

Human eating behavior is not only guided by a conscious and reflective system, but also via a non-conscious, impulsive, associative system that is susceptible to food promotion techniques [20, 46–50]. By using different food promotion interventions within the vlogs, like using unboxing, preparing and taste testing the fruits and vegetables (taste) versus using a challenge (humor and fun) [51] to increase the rewarding value of fruits and vegetables, it will be possible to test differences between media messages [19, 20, 52–67]. The current project proposes that food promotion techniques increase the reinforcing value of fruits and vegetables among young children, which in turn has a positive effect on the immediate intake.

#### H1

The promotion of fruit and vegetables will increase the reinforcing value of fruits and vegetables.

#### H2

The reinforcing value of fruit and vegetables has a positive and reciprocal effect on the immediate intake of fruit and vegetables.

### Long-term effects: habitual formation

Most studies have examined immediate or short-term effects of healthy food promotion techniques [65–68], while long-term effects are particularly important for vital health improvements [1–9]. Numerous studies have shown that repeatedly exposing and tasting fruit and vegetables increases the liking of it, thereby increasing the probability of consumption [69–74]. Availability and mere exposure are processes by which experiences of a stimulus positively enhances the attitudes towards it, resulting in the given stimulus acquiring positive valence and becoming more apparent in the mental scheme of children [69, 75], thereby habitualizing the consumption of fruit and vegetables [74, 75] and possibly even substituting the HFSS foods by healthier snacks [53, 74–78].

#### H3

The intake of fruit and vegetables on the short term will lead to habitual forming that will lead to an increased fruit and vegetable intake on the long-term.

### Individual and contextual susceptibility

Multiple individual and contextual factors have been established that explain susceptibility to food promotion of unhealthy food [20, 24]. For example, age [42, 43], impulsivity [79], weight status [42, 43] and attentional bias [80] have been found to moderate the effect of unhealthy food promotion on children’s intake, but I will investigate whether these concepts moderate the effect of promotion of fruit and vegetables. Other individual factors that are distinguished as possible moderating factors are neophobia and food fussiness [44, 45]. Additionally, contextual factors, like parental feeding techniques [46, 47] or social economic status [81, 82] are highly related to the intake of fruit and vegetables. Establishing individual and contextual dispositional factors, are vital for our understanding the variability in the processing of the promotion of fruit and vegetables, the reinforcing value of these foods, and subsequent intake.

#### H4

Individual and contextual susceptibility factors will moderate the effects of food promotion techniques of fruit and vegetables on intake.

## Methods/design

The main aim of the current study is to test a newly designed overarching theoretical model that explains and predicts whether, how, when, and for whom food promotion techniques increase children’s fruit and vegetables intake, both on the short- and long-term [82]. Three separate studies will be conducted to test this: (1) focus groups, (2) multicenter randomized clinical trial, and (3) follow-up measurements. All participants in the three studies will provide written consent for participation to the project leader. These studies will be conducted in the Netherlands, starting in April 2020 and ending in December 2022.

### Study 1: focus groups

First, to develop the vlogs in co-creation different focus groups will be held with (1) children (*N* = 25, between 8 and 13 years), (2) parents (*N* = 10), (3) vloggers (*N* = 5), and (4) fruit and vegetable producers and marketers (N = 5). The main question for the focus groups are: “What should be the exact content and methods of the vlog to increase (which) fruits and vegetables among the target group?”. The intervention materials will be pilot-tested among the target group (*N* = 100). The primary outcomes from these focus groups will be used to develop the exact intervention.

### Study 2: conduct intervention (T1 and T2)

Second, participating primary schools (from low-, middle- and high-SES areas) in the randomized controlled experimental intervention-study (computer-generated randomization) will receive a food stand located on a key-point in school, with standard fresh, free, and ready-to-eat fruit and vegetables that can be consumed during the breaks [63]. The number of children that consume fruit and vegetables will be counted (with a clicker [63]) and calculated afterwards by the experimenter and research assistant. Children in the experimental condition will watch the promotional vlog (10–20 min) three times a week. A mixed repeated measure design with three different conditions will be used: (1) control, (2) a vlog unboxing fruit and vegetables (preparing and tasting), and (3) a vlog doing a challenge with the fruit and vegetables (e.g., contests, tricks, games). Written consent from the principal and teachers will be obtained first, followed by written consent from parents and verbal consent from children. Children can stop participation during the experiment without any reason. Collected data from this participants will be deleted. All collected data will be pseudonymized, whereas anonymized data will be made available for other researchers. Randomization will take place at school level.

**Fig. 1:**
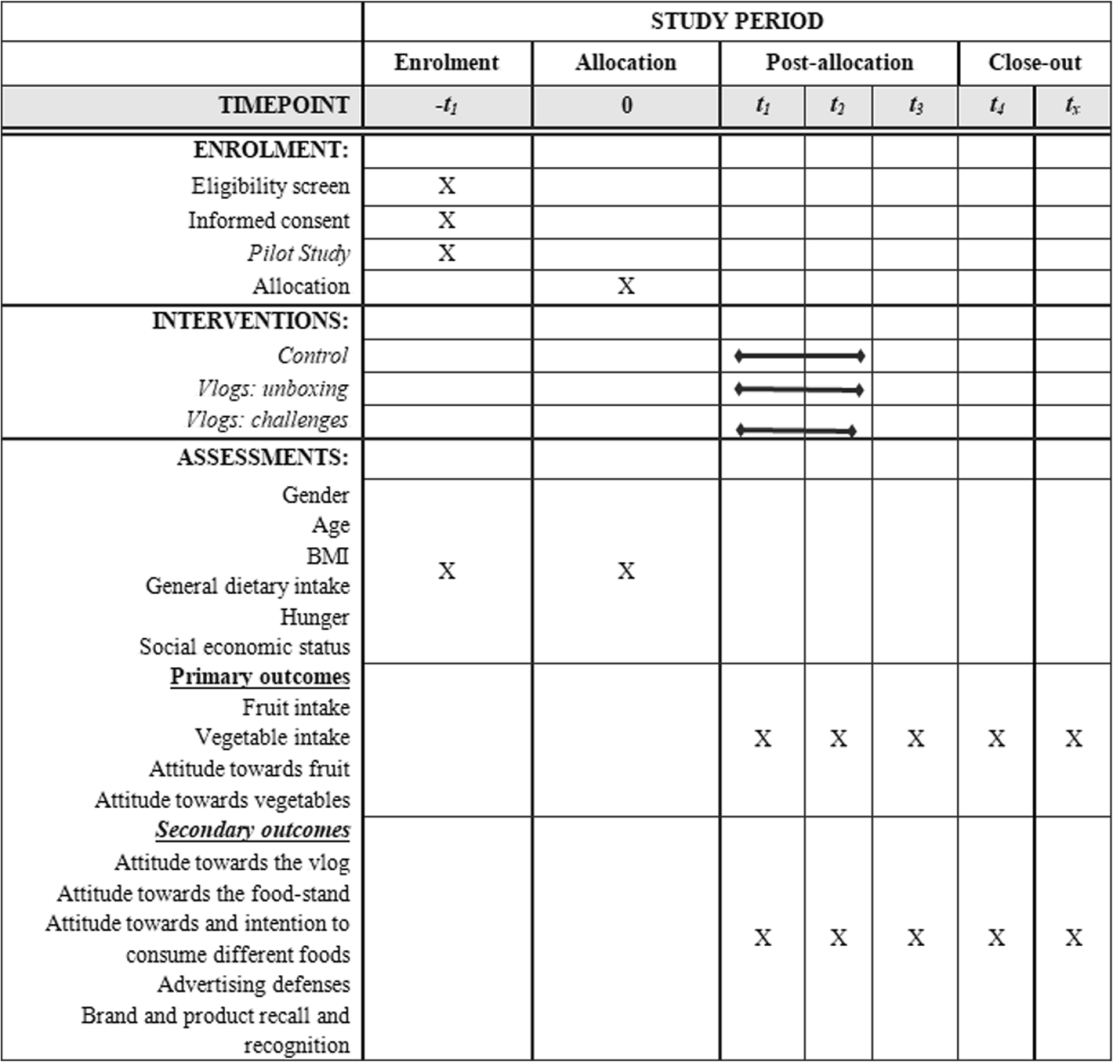
Flow schedule of enrolment, interventions, and assessments of the randomized controlled trial and follow-up measurements (Study 2 and 3)

Power analyses (G*Power) [83] suggest that *N* = 342 is expected to be sufficient to detect significant (α = 0.05), medium effects (f = 0.25) of conditions (power = 0.80). Next, children will complete questionnaires including pre- and post-test measurements during school hours together with the experimenter to assess general dietary intake [13], before (baseline, T1) and immediately after the intervention (T2, 10-weeks after the baseline, see Fig. 1). Primary outcome of this study is the intake of fruit and vegetables. Secondary outcomes are liking and wanting of fruit and vegetables [28], attitudes towards the vlog [79, 80], attitudes towards the food-stand [79, 80], attitudes towards and intention to consume different foods (e.g., fruit, vegetables, HFSS foods) [79, 80], advertising defenses [79, 80], brand and product recall and recognition [79, 80]. These outcomes will be assessed via questionnaires together with the experimenter. Children’s age, hunger [79, 80], sex, SES [12, 13], and BMI [79, 80] will be included as control or moderating variables. All measures have been validated among this age group in previous research.

### Study 3: follow-up measurements (T3 and T4)

After 6 (T3) and 12 months (T4) follow-up measurements will take place. Actual intake during lunch in schools, liking of fruit and vegetables, and attitudes towards and intention to consume foods, will be assessed again during school time.

### Recruitment

Different primary schools have already agreed on participation (*N* = 4). Based on the existing network of the researcher, additional schools will be targeted after focus groups have been conducted.

### Data analysis

Bayesian mixed effects models will be carried out in R using the brms package [84] to examine differences in all dependent and mediating constructs between pre- and post-test conditions. In addition, multilevel regression analyses will be conducted to examine possible group differences. Structural Equation Modeling will be used to examine whether the liking and wanting of fruits and vegetables mediates the effect of the promotion of fruit and vegetables on the intake.

## Discussion

This study systematically tested whether a healthy food promotion intervention was effective in increasing actual fruit and vegetable intake among children. The study will provide highly relevant, valid and reliable information about the effectiveness of a persuasive instrument. The current study is highly original and innovative as it is the first that systematically examines the influence of food promotion on actual intake of fruit and vegetables [24]. In addition, it assesses repeatedly intake both on the short- and long-term, validates a newly developed theoretical framework to establish the underlying mechanism, focuses on vlogs as a persuasive instrument, and will take into account intensively on individual susceptibility factors.

Considering the design of the study, with primary schools, teachers and children actively involved, some practical and operational issues involved in performing the study are important to discuss here. First, active consent from parents is needed for participation of children. Taking into account that this will be a longitudinal study, the drop-out rate is something importantly that must be well-thought-out. For example, including multiple schools will increase the probability that enough children will participate in the experimental study to conduct statistical analyses with enough statistical power. Therefore, the aim is to include more than 10 schools to make sure enough children can participate.

Second, using co-creation has been proven to be a very effective methodology to create innovations [85], by increasing external and ecological validity. To make this work, structural and active participation of the participation is needed [86]. Therefore, structured focus groups need to be organized and planned in advance, and different stakeholders need to be involved to get a more profound understanding from all the various perspectives.

Third, other factors might be involved in influencing the intake of fruits and vegetables, for example if children have the means to purchase the food outside the school and what parents provide their children with to eat during school. This will be overcome because on the short-term, effects on the consumption of fruits and vegetables in primary school settings during school time will be assessed, where purchase of other foods in schools is simply not possible in the Netherlands. Next, to establish the intervention effects in other situations outside school, I will assess long-term intake by repeated assessment through questionnaires. Furthermore, because the fruits and vegetables will be provided at school, parents will be informed that they should not provide their children with fruit and vegetables, which is similar to current EU-school fruit projects on primary schools.

Final, considering that a large amount of data will be collected among young children, a specific data management plan and strict protocol has been written in close collaboration with the data steward of the host institute (Tilburg School of Humanities and Digital Sciences, the Netherlands). The host institute is very strict in the treatment of the collected data with the highest level of confidentiality to assure good management of data.

## Data Availability

The datasets used and/or analysed during the current study are available from the corresponding author on reasonable request. In order to protect privacy, personal information will be pseudonymized for the project leader and anonymized for other researchers. Data will be stored in a dark archive of the Tilburg University.

## Abbreviations

BMI: Body Mass Index
HFSS: High in Fat, Salt and Sugar
SES: Social Economic Status
